# Error Characteristic Analysis and Filtering Algorithm for GNSS Time-Series Data

**DOI:** 10.3390/s25020361

**Published:** 2025-01-09

**Authors:** Hongli Zhang, Yijin Chen, Kemeng Li, Yinggang Wang

**Affiliations:** College of Geoscience and Surveying Engineering, China University of Mining and Technology (Beijing), Beijing 100083, China; bqt2100205061@student.cumtb.edu.cn (H.Z.); bqt2200205054@student.cumtb.edu.cn (K.L.); bqt2200205055@student.cumtb.edu.cn (Y.W.)

**Keywords:** time series data, filtering algorithm, error compensation

## Abstract

Under regional environmental conditions such as open-pit mines and construction sites, there are usually fixed GNSS measurement points. Around these fixed stations, there are also mobile GNSS measurement modules. These mobile measurement modules offer advantages such as low power consumption, low cost, and large data volume. However, due to their low accuracy, these modules can only provide approximate positions as monitoring data, such as for vehicle management in open-pit mines. To extract more information from the existing large volume of low-accuracy data, it is necessary to process these low-accuracy data. Under conditions of the same time and space in a small area, factors affecting measurement accuracy can be comprehensively considered. By analyzing the temporal GNSS data within the same spatiotemporal small region and understanding the variation patterns of measurement errors, a general equation for measurement error variation can be formulated. Using filtering methods, the data quality can be improved. Through the analysis of the experimental data in this study, it was found that the variation patterns of measurement data obtained by devices of the same accuracy during the same time period are generally consistent. After applying filtering methods, the measurement accuracy of each station improved by up to approximately 95.9%, with a minimum improvement of approximately 84.4%. Under the condition of a 95% confidence level, the reliability increased by up to approximately 73.2%, with a minimum improvement of approximately 58.2%. These experimental results fully demonstrate that under regional spatiotemporal conditions, the temporal data obtained by GNSS measurement devices with similar accuracy exhibit similar error distribution patterns. Applying the same filtering method can significantly improve the accuracy and reliability of measurement data.

## 1. Introduction

In recent years, the widespread application of Global Navigation Satellite System (GNSS) technology [1] has played an important role in measurement and monitoring under regional environmental conditions such as open-pit mines and construction sites. Examples include open-pit mine vehicle management, monitoring the locations of construction machinery and materials, and individual or group positioning [2,3,4]. In these scenarios, portable positioning modules are often surrounded by fixed GNSS stations. Some measurement processes require the collaboration of both fixed and mobile stations. However, in most application scenarios, low-cost GNSS positioning modules cannot interact with the high-precision data from fixed stations [5,6,7,8]. Due to their low power consumption, affordability, and compact size, mobile GNSS modules are widely used, resulting in an enormous amount of data [9,10,11]. Because of their low cost, these devices are more susceptible to environmental influences such as multipath effects, signal blockages, and electromagnetic interference in complex measurement environments, leading to significant fluctuations in data quality [12,13,14,15]. These issues significantly reduce the value of GNSS module data, which is characterized by high spatial density and large data volume. Effectively processing and analyzing existing massive low-accuracy data to improve its precision and reliability have become a pressing issue [16,17,18,19]. Under the same temporal and small-area conditions, factors affecting GNSS measurement accuracy—such as satellite geometry, environmental obstructions, and signal quality—are consistent [20,21,22]. By analyzing GNSS time-series data under identical conditions, the variation patterns of measurement errors can provide critical guidance for data processing and accuracy improvement. Based on this theoretical foundation, this experiment conducted an in-depth analysis of GNSS time-series data within the same spatiotemporal region to reveal the variation patterns of measurement errors [23,24]. In practical applications, filtering methods are widely used to optimize measurement data. By identifying and compensating for measurement error variation patterns, the quality of low-accuracy data can be significantly improved [25,26]. By comprehensively considering various factors affecting measurement accuracy, this study modeled time-series data errors and proposed corresponding improvement measures [27].

In the experiment, filtering methods were applied to GNSS time-series data from devices with similar accuracy levels, and the results demonstrated a significant improvement in data quality. The experimental results showed that, after applying filtering methods, the measurement accuracy of each station improved by 84.4% to 95.9%, and reliability under a 95% confidence level increased by 58.2% to 73.2%. These results fully demonstrate that filtering methods based on error variation analysis can significantly enhance the accuracy and reliability of GNSS measurement data, providing a feasible solution for the in-depth utilization of low-accuracy data.

Based on these findings, this study aims to simulate data from low-precision modules. By analyzing the error patterns in low-precision time-series data and applying filtering techniques, we seek to further enhance data quality. This research intends to provide theoretical support and practical guidance for GNSS data applications in related scenarios, such as open-pit mines. It not only expands the theoretical foundation for improving GNSS data quality but also offers technical support for practical applications. By systematically analyzing the characteristics of GNSS time-series data under the same spatiotemporal conditions, this study proposes a filtering method based on error time-series patterns, offering new solutions and approaches for these application scenarios. With the continuous optimization of GNSS data processing algorithms, the potential of low-cost, low-accuracy GNSS modules will be further realized, and the value of regional GNSS data will be more fully utilized.

## 2. Materials and Methods

**Figure 1:**
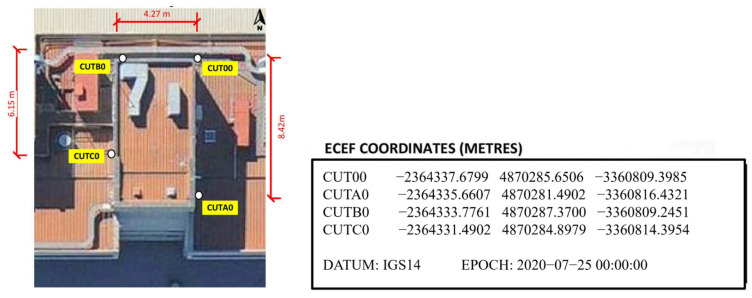
Distribution and true coordinates of experimental data stations.

Several fixed stations’ data were used to analyze short-term time-series measurements. When processing data from these fixed stations, a single-point positioning mode similar to that of single-point positioning modules was applied. The data source is observational data from Curtin University in Australia (https://gnss.curtin.edu.au/, accessed on 25 October 2023), with fixed stations CUT00, CUTA0, CUTB0, and CUTC0. The station distribution map along with the true coordinates of each station is presented in Figure 1.

**Figure 2:**
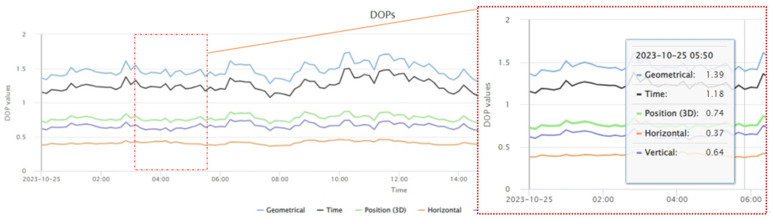
Diagram of satellite DOP value changes on 25 October 2023.

**Figure 3:**
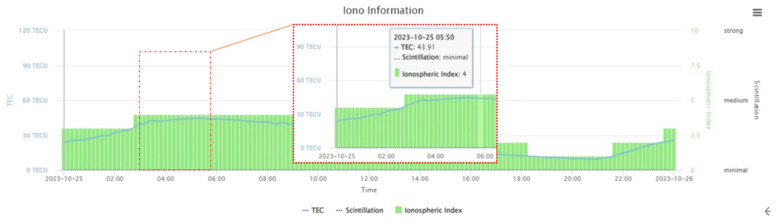
Diagram of ionospheric variations on 25 October 2023.

The antenna model for the above stations is TRIMBLE TRM 59800.00 SCIS, mounted on steel masts. Each antenna is connected to three high-quality geodetic receivers via a signal distributor, with each of the four stations having a different receiver. These are multi-frequency, multi-system GNSS receivers that provide GPS, GLONASS, GALILEO, QZSS, and BDS data, with a sampling interval of 30 s in RINEX 3.x format. The data used for this experimental analysis is observational data from 25 October 2023 (Julian Day 298). Based on satellite DOP values and ionospheric data from that day, the specific time range for the data is 03:40–05:50. Satellite DOP values and ionospheric indices are two important indicators in calculating measurement coordinates. The changes in these two factors on 25 October 2023 are shown in Figure 2 and Figure 3 as follows:

By analyzing publicly available data such as GNSS module measurement data, this study effectively demonstrates the randomness of the experiment and the general applicability of the filtering algorithm. By calculating the real-time coordinates of the four stations, the measurement error variation over time is observed and analyzed, and a filtering algorithm for correcting measurement errors is derived. The downloaded raw files were decompressed into RINEX format o and n files, and RTKLIB was used for processing. To simulate the positioning mode of commonly used low-cost GNSS modules, the single mode was selected in the RTKPOST module, which provided real-time coordinates for the CUT000, CUTA00, CUTB00, and CUTC00 stations on 25 October 2023. The experiment primarily analyzes coordinate errors from short time series (within half an hour). Therefore, we need to extract the .pos data for the 24 h period after processing. Two main factors were considered when selecting the data: DOP values and ionospheric index. Data with a DOP value of <2 and a stable ionospheric index were selected, with the time range for extraction being from 03:40 to 05:50 on 25 October 2023.

**Figure 4:**
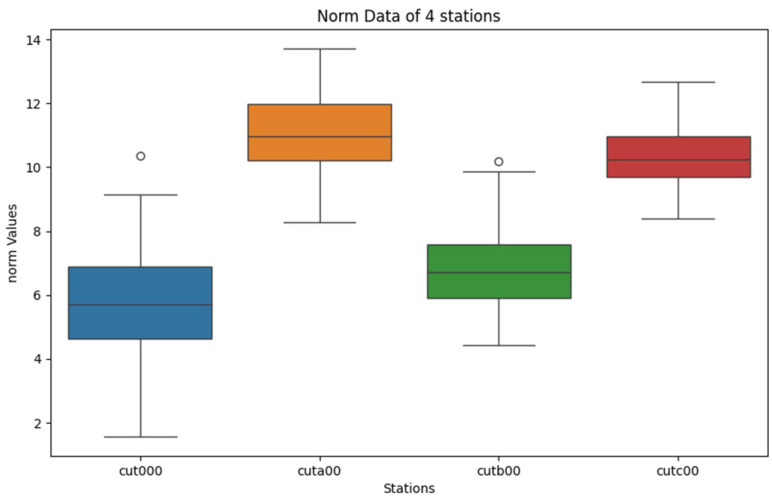
Box plot of measured errors for the four stations.

Low-cost GNSS modules typically provide real-time latitude and longitude coordinates in their NMEA data stream. However, the data in this experiment are raw measurement data, which need to be processed to obtain the measurement point coordinates. The coordinates were calculated from the observed raw data, and these calculated coordinates were treated as the real-time positioning information for analysis. The differences between the observed values and the true coordinate values were used to provide the basis for the filtering algorithm. The coordinates obtained using RTKPOST software (v2.4.3 b34) were in the WGS-84 format, and Python was used to batch-convert the coordinates into ECEF coordinates. The real-time coordinates $\left(x_{t},\;y_{t},\;z_{t}\right)$ calculated from the real-time observation data differ from the true coordinates $\left(x,\;y,\;z\right)$ at each time point. The norm of the 3D coordinate differences at each time point is calculated, which represents the measurement error. The measured errors for each measurement point are displayed using a box plot to analyze the overall distribution of the measurement errors. The box plot is shown in Figure 4 as follows:

From the box plots of the four stations, it can be observed that the data dispersion is essentially consistent across all stations, with almost no outliers. There is a difference in the central values of each station, which aligns with the fact that different measurement equipment was used at each station. This also indicates that the positional errors caused by different receivers are quite significant. This result provides insights for data analysis using the same type of receiver equipment.

Several factors affect the accuracy of single-point positioning, including satellite ephemeris errors, atmospheric delays, satellite clock biases, and so on. Differential GPS (DGPS) is a common method of enhancing single-point positioning accuracy. When two stations with good spatiotemporal correlation (i.e., located relatively close to each other) are equipped with GNSS receivers performing single-point positioning simultaneously, the aforementioned errors impact both stations similarly. If one receiver is placed at a known point, comparing its calculated position with the known coordinates reveals the influence of these errors on the station’s position.

In practical applications, DGPS transmits the determined correction values to nearby users via data communication links. By applying these corrections, users can significantly improve their positioning accuracy. Common differential corrections include position and range corrections. For low-precision GNSS modules that provide direct position information, applying error compensation through filtering, based on DGPS principles, is a feasible data processing approach to enhancing positioning accuracy.

**Figure 5:**
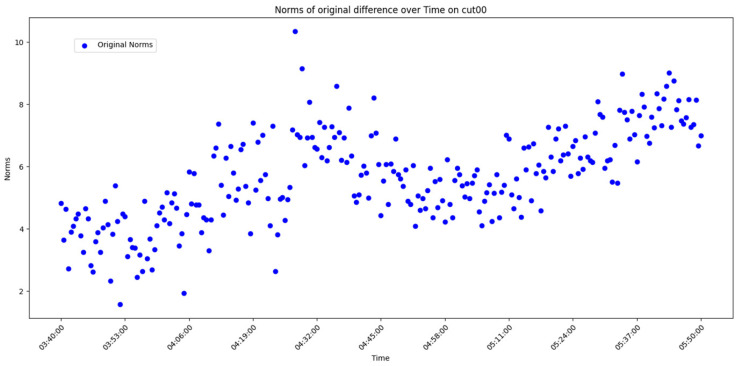
Diagram of measured error distribution over time for station CUT00.

**Figure 6:**
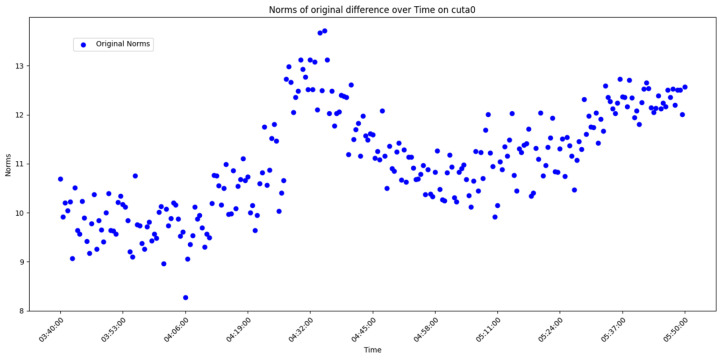
Diagram of measured error distribution over time for station CUTA0.

**Figure 7:**
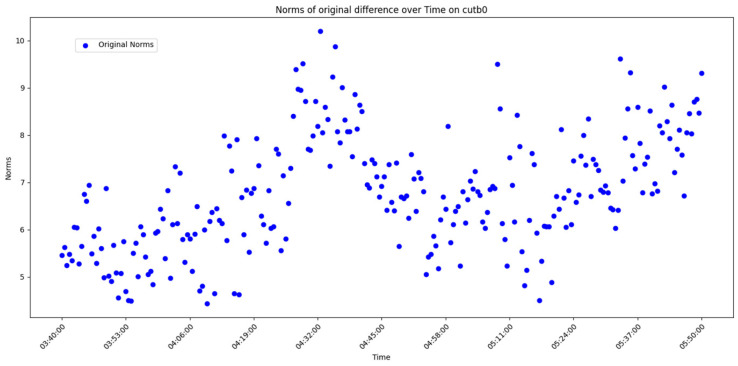
Diagram of measured error distribution over time for station CUTB0.

**Figure 8:**
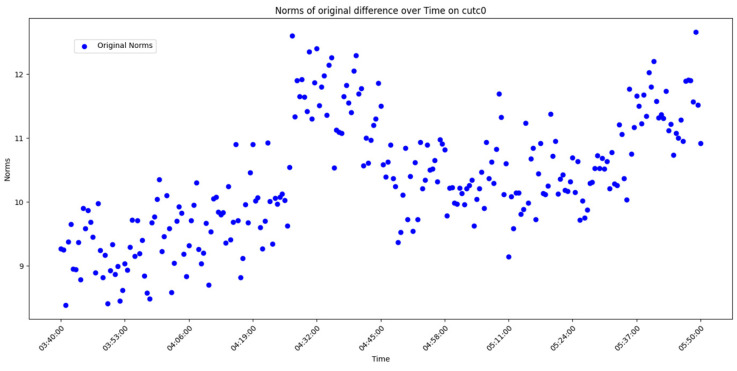
Diagram of measured error distribution over time for station CUTC0.

After overall comparative analysis, the measured errors for each station were arranged in chronological order, yielding a distribution chart showing how the measured errors change over time, as shown in Figure 5, Figure 6, Figure 7 and Figure 8:

We are aware that factors affecting GNSS measurement accuracy mainly include equipment performance, environmental conditions, computational methods, etc. Under conditions of a small area and short duration, we analyze all influencing factors as a whole. The “original norms” in the above image refer to the norm of the difference between the true coordinates and the real-time calculated coordinates, representing the actual measurement error. By observing and analyzing the measured error distribution over time for each station, we can see that around 04:32:00, all the measurement devices show a peak in their data. Looking at the overall trend, the variation trend of the norm of the difference between the true values and real-time measurement values for all measurement devices is also quite consistent. Here, we do not discuss the cause of the peak or the factors affecting the change in measured error. The phenomenon we focus on is that, under relatively stable temporal and spatial conditions, when considering all the factors that may influence measurement accuracy together, the GNSS measurement error is a time-varying quantity or, more specifically, a quantity related to the measurement sequence. Thus, we can analyze the measurement errors using time-series methods.

## 3. Results

For analyzing time-series data, trend-reflecting methods are well suited for this experiment. Polynomial regression is used to describe the variation in measured errors, and residual analysis is applied to filter the deviation between the polynomial regression predictions and actual values. This process yields filtered coordinate values with significantly improved accuracy.

### 3.1. Establish a Filtering Model

#### Multivariate Polynomial Regression Algorithm

In nature, objective phenomena are always closely interconnected, interdependent, and mutually influential. These complex relationships can generally be classified into two types. The first type is a deterministic relationship, where there is a clear functional relationship between the dependent and independent variables. The second type is an indeterminate dependency relationship, where the variables are clearly related but cannot be expressed using an exact function. Regression analysis is a statistical method used to handle such relationships between variables.

In this experiment, all possible errors affecting measurement accuracy are treated as a whole. Clearly, this “overall error” inevitably affects the measurement results, but the specific functional relationship between the two cannot be determined. Furthermore, for time-series data, analytical methods that reflect trend changes can better preserve the temporal characteristics of the time-series data.

The Multiple Polynomial Regression model can capture the influence of multiple independent variables on a target variable and is widely used for modeling and prediction in various scientific, engineering, and statistical fields [28].

**Figure 9:**
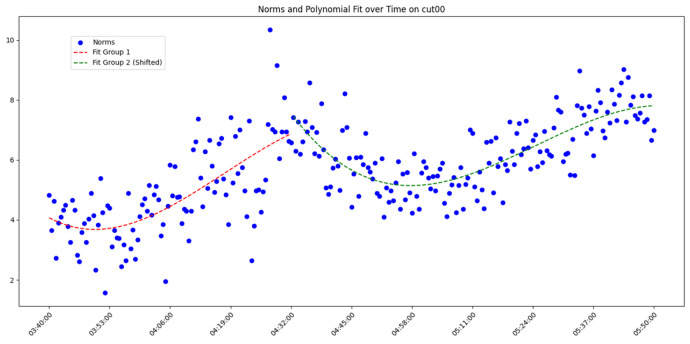
Diagram of measured errors and fitted curve for station CUT00.

**Figure 10:**
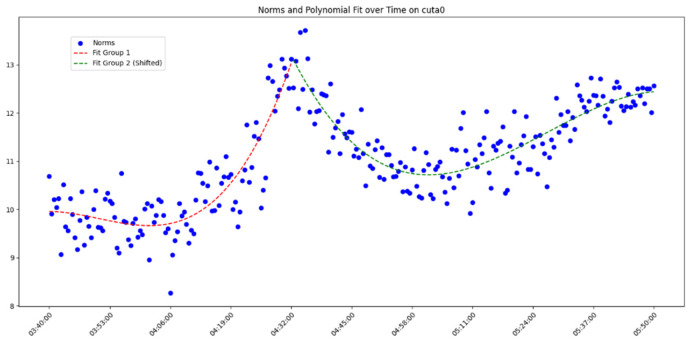
Diagram of measured errors and fitted curve for station CUTA0.

**Figure 11:**
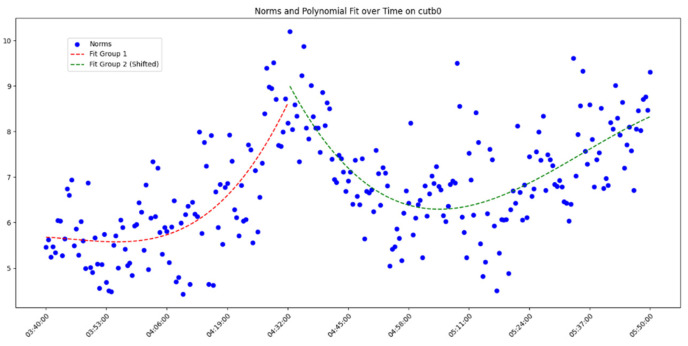
Diagram of measured errors and fitted curve for station CUTB0.

**Figure 12:**
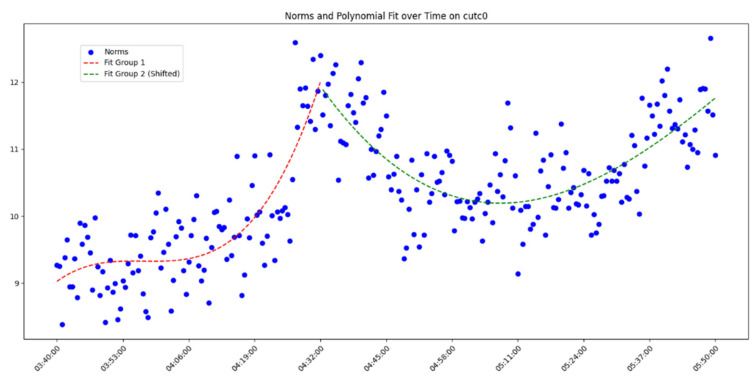
Diagram of measured errors and fitted curve for station CUTC0.

In analyzing the time-series variation in measured errors shown in the above process, we found that a trivariate quadratic regression model fits well in describing the pattern of measured error changes. The fitted curve and the actual measured errors are displayed together in a single graph, with the results shown in Figure 9, Figure 10, Figure 11 and Figure 12 as follows:

Since each station exhibits a peak, fitting the data across the entire time period would include significant noise and is therefore unreasonable. Instead, we opted to group the data and perform fitting after the data segmentation. At the peak near 04:32:00, the data were divided into two groups. Clearly, as shown in the figure, the cubic quadratic regression model performs exceptionally well. Regardless of the receiver model at the station, the fitting equation accurately captures the variation in the observed error over time. This indicates that under consistent computational methods and spatial conditions, the measurement errors produced by different receiver models (which differ only in model but maintain the same measurement accuracy) vary over time, and this variation can be expressed using a universal model. Consequently, the errors expressed by the universal equation can be treated as noise and filtered. The observed errors after being filtered using the regression equation are referred to here as error residuals, representing the residuals between the observed errors and their predicted values.

### 3.2. Residual Analysis

Residual analysis is an evaluation method for model fitting results, used to measure the deviation between the model’s predicted values and the actual observed values. Residual analysis helps detect the model’s suitability, diagnose whether there are systematic errors in the model, and assist in improving the model’s accuracy [29,30,31].

1. Definition of Residuals
  A residual is the difference between the actual observed value and the model’s predicted value. For an observed value $n_{i}$ and its corresponding predicted value $\hat{n_{i}}$, the residual $e_{i}$ is defined as

(1) $$e_{i}=n_{i}-\hat{n_{i}},$$

where

$n_{i}$ is the *i*-th actual observed value;

$\hat{n_{i}}$ is the predicted value of the *i*-th observation from the regression model.

In the experiment of this paper, $n_{i}$ is the norm of the difference between the measured coordinates $\left(x_{t},y_{t},z_{t}\right)$ and the true coordinates $\left(x,y,z\right)$, and $\hat{n_{i}}$ is the predicted value of the *i*-th norm.

2. Steps of Residual Analysis

Step 1: Calculate Residuals

Using the formula above, calculate the residuals corresponding to all observation values. Each data point has a residual $e_{i}$, resulting in the residual sequence $\left\{e_{1},e_{2},\ldots ,e_{n}\right\}$.

Step 2: Visualization of Residuals

Use graphical methods to examine the distribution and characteristics of the residuals. In this experiment, the measured errors of each station over time have already been displayed (Figure 5, Figure 6, Figure 7 and Figure 8). To visualize the difference between actual measurement errors and their predicted values (i.e., error residuals), a line chart displaying changes over time can be employed. Additionally, a simple linear regression equation ACF plot can be utilized to depict the temporal variation pattern of these error residuals.

Step 3: Statistical Analysis of Error Residuals

Use statistical measures to analyze the characteristics of the error residuals, commonly including the following:

Mean: Theoretically, the mean of the residuals should be close to zero. If the mean residual deviates from zero, it indicates a systematic bias in the model.

(2) $$\bar{e}=\frac{1}{n}\sum_{i=1}^{n}\;e_{i},$$

Variance: The variance in the residuals is used to assess the model’s prediction error. The smaller the residual variance, the better the model fit.

(3) $$Var(e)=\frac{1}{n}\sum_{i=1}^{n}\;(e_{i}-\bar{e}{)}^{2},$$

Mean Squared Error (MSE): A commonly used metric for measuring the model’s fitting error. MSE calculates the sum of the squares of each residual and then takes the average.

(4) $$MSE=\frac{1}{n}\sum_{i=1}^{n}\;(e_{i}{)}^{2},$$

Standard Error (SE): The standard error is the standard deviation of the residuals, used to assess the dispersion of the residuals.

(5) $$SE=\sqrt{\frac{1}{n}\sum_{i=1}^{n}\;e_{i}^{2}},$$

In this experiment, the commonly used root-mean-squared error (RMSE) is employed to evaluate the performance of the regression model.

1. Common Tools for Residual Analysis

Residuals in Linear Regression: In linear regression models, residual analysis can be used to check if the linear assumptions hold. The residuals should be independent and identically distributed, following a normal distribution.

Residuals in Time Series: In time-series analysis, residuals can be used to assess how well the model fits dynamic data. For example, in an ARIMA model, the residuals should resemble white noise, with no significant autocorrelation.

**Figure 13:**
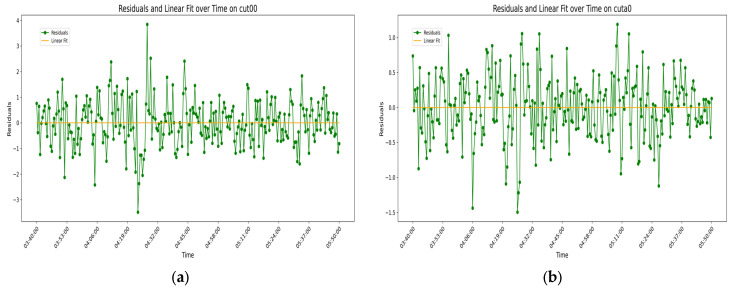

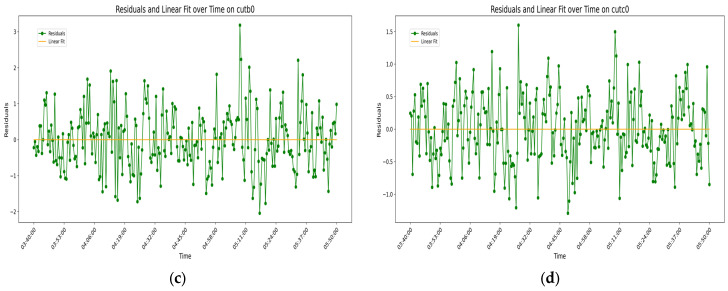
The line in the above image represents the variation in station error residuals over time, while the nearly horizontal line is the simple linear regression graph of the error residuals: (**a**) time series of error residuals and their simple linear regression for station CUT00; (**b**) time series of error residuals and their simple linear regression for station CUTA0; (**c**) time series of error residuals and their simple linear regression for station CUTB0; (**d**) time series of error residuals and their simple linear regression for station CUTC0.

**Figure 14:**
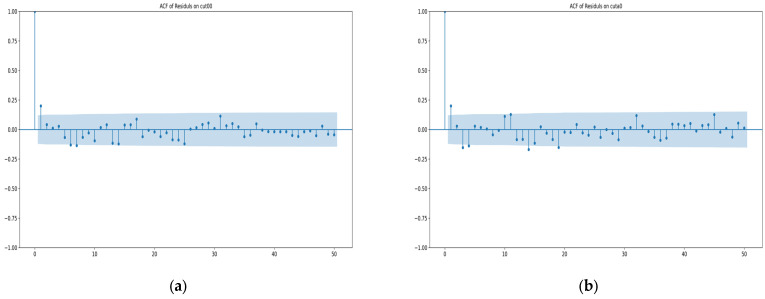

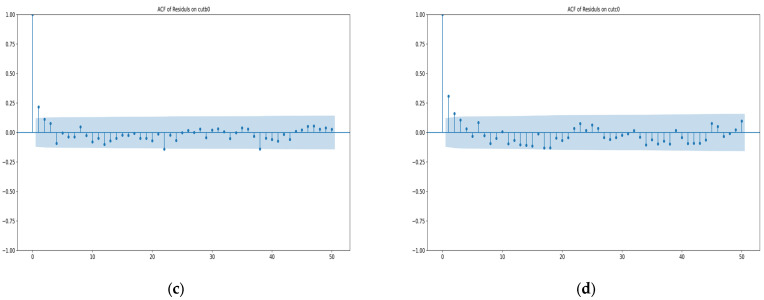
In the above images, the horizontal axis represents the lag order, the vertical axis represents the autocorrelation coefficient, and the blue shaded area indicates the confidence interval: (**a**) ACF graph of error residuals for station CUT00; (**b**) ACF graph of error residuals for station CUTA0; (**c**) ACF graph of error residuals for station CUTB0; (**d**) ACF graph of error residuals for station CUTC0.

This experiment analyzes the error residuals of the time series, and the variation pattern of the error residuals over time is shown in Figure 13 as follows:

From the figure, it can be seen that the variation in error residuals over time is irregular. The simple linear regression graph for the residuals of each station is a line close to zero, indicating that time-related errors have essentially been eliminated, and the error residuals are random errors. To further demonstrate the randomness and stochastic nature of the error residuals, the ACF (autocorrelation function) graphs of the error residuals for each station are plotted in Figure 14 as follows:

From the ACF graphs of the error residuals for the four stations above, the following observations can be made:

a. Near lag 0, the autocorrelation coefficient is 1 for all stations, indicating that the data are completely self-correlated, reflecting the characteristics of random errors.

b. For other lag orders, the autocorrelation coefficients are mostly randomly dis-tributed within the confidence interval and close to 0, which is consistent with the characteristics of random errors.

c. The ACF graphs do not exhibit any regular oscillations (e.g., sinusoidal or exponential decay patterns), suggesting that the data do not have significant periodicity or trends, which aligns with the properties of random errors.

From the above analysis, it can be seen that the variation in error residuals over time for each station conforms to the behavior of random errors. From the data characteristics reflected in the two sets of figures, it can be concluded that the filtering model for measurement error variation over time truly exists and that the cubic multiple regression model performs well in capturing the generality of the variation in measurement errors over time.

### 3.3. Model Evaluation

**Figure 15:**
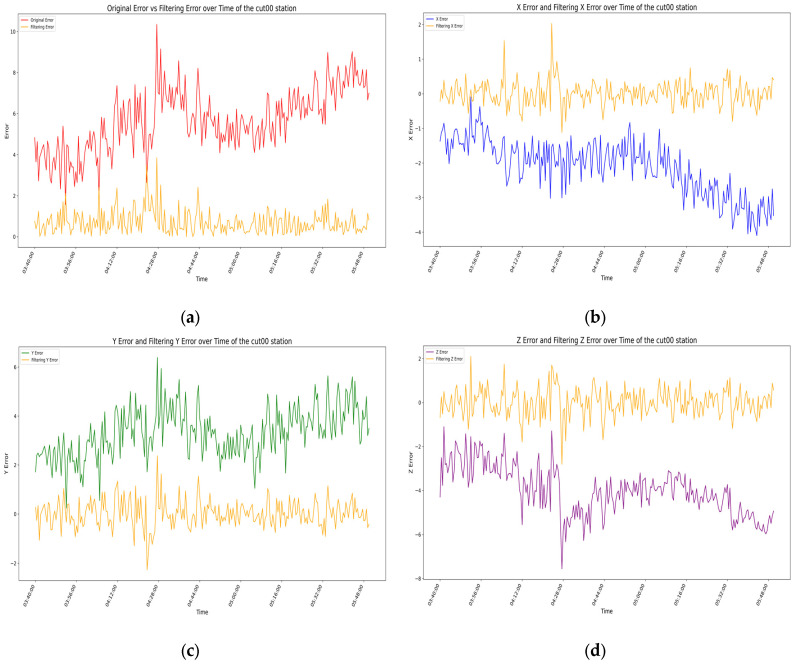
In the above image, the orange curve represents the filtered data variation for the CUT00 station, while the other colors represent the data variation before filtering: (**a**) the variation in station errors before and after filtering at the CUT00 station; (**b**) the variation in X-direction errors before and after filtering at the CUT00 station; (**c**) the variation in Y-direction errors before and after filtering at the CUT00 station; (**d**) the variation in Z-direction errors before and after filtering at the CUT00 station.

**Figure 16:**
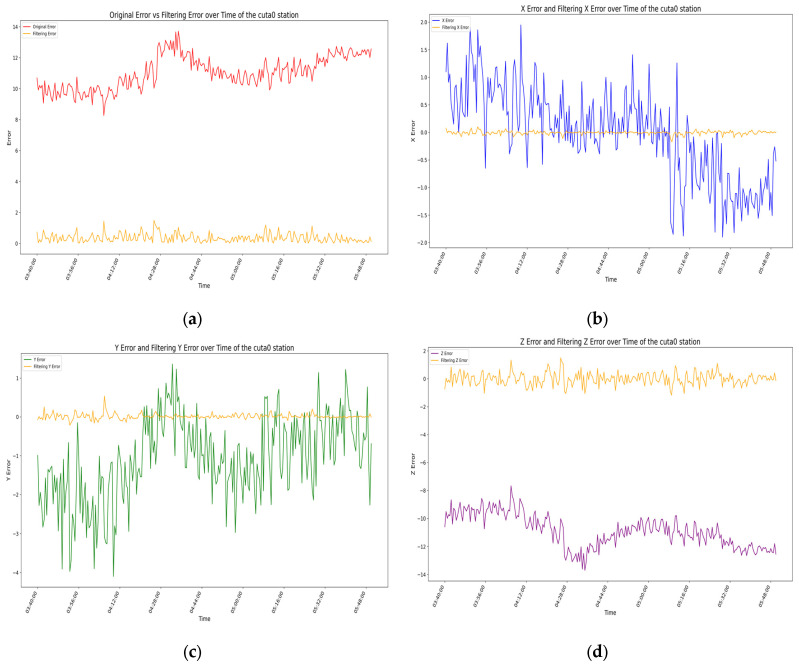
In the above image, the orange curve represents the filtered data variation for the CUTA0 station, while the other colors represent the data variation before filtering: (**a**) the variation in station errors before and after filtering at the CUTA0 station; (**b**) the variation in X-direction errors before and after filtering at the CUTA0 station; (**c**) the variation in Y-direction errors before and after filtering at the CUTA0 station; (**d**) the variation in Z-direction errors before and after filtering at the CUTA0 station.

**Figure 17:**
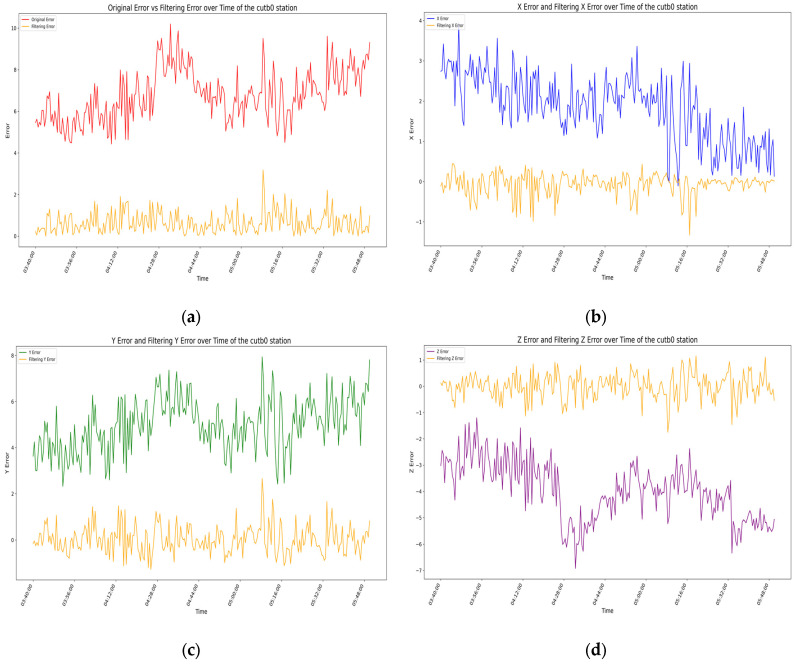
In the above image, the orange curve represents the filtered data variation for the CUTB0 station, while the other colors represent the data variation before filtering: (**a**) the variation in station errors before and after filtering at the CUTB0 station; (**b**) the variation in X-direction errors before and after filtering at the CUTB0 station; (**c**) the variation in Y-direction errors before and after filtering at the CUTB0 station; (**d**) the variation in Z-direction errors before and after filtering at the CUTB0 station.

**Figure 18:**
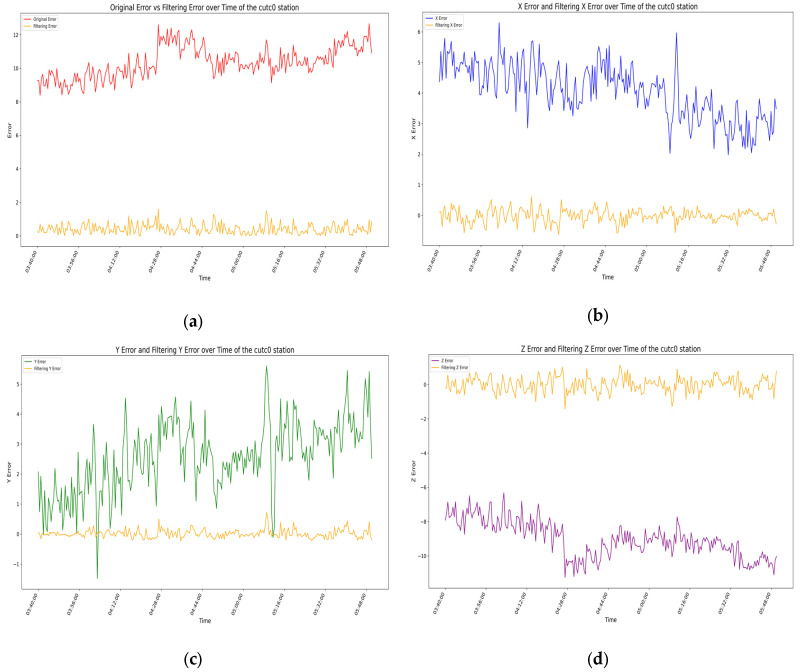
In the above image, the orange curve represents the filtered data variation for the CUTC0 station, while the other colors represent the data variation before filtering: (**a**) the variation in station errors before and after filtering at the CUTC0 station; (**b**) the variation in X-direction errors before and after filtering at the CUTC0 station; (**c**) the variation in Y-direction errors before and after filtering at the CUTC0 station; (**d**) the variation in Z-direction errors before and after filtering at the CUTC0 station.

For the above filtering model, by analyzing the error residuals, we know that the model has good generality, but the specific expression effect needs further analysis. We evaluate the filtering effect of the prediction model by analyzing the root-mean-square error (RMSE) of the measurement coordinates before and after filtering [32,33], with the results for each station shown in Figure 15, Figure 16, Figure 17 and Figure 18 as follows.

The root-mean-square error (RMSE) of the measurement data and the confidence interval at the 95% confidence level before and after filtering for the CUT00 station are shown in Table 1 below:

The root-mean-square error (RMSE) of the measurement data and the confidence interval at the 95% confidence level before and after filtering for the CUTA0 station are shown in Table 2 below:

The root-mean-square error (RMSE) of the measurement data and the confidence interval at the 95% confidence level before and after filtering for the CUTB0 station are shown in Table 3 below:

The root-mean-square error (RMSE) of the measurement data and the confidence interval at the 95% confidence level before and after filtering for the CUTC0 station are shown in Table 4 below:

In the four figures for each station, the total error and the error variations in the X-, Y-, and Z-directions before and after filtering are presented. Through comparative analysis of these four figures, it is evident that the total error and the errors in each direction exhibit significant variations over time before filtering, with considerable uncertainty in the range of errors. By analyzing the temporal variations in errors at the aforementioned stations, it is evident that, whether considering the total error or the individual directional errors, the filtered changes are fundamentally consistent and uniform. Before filtering, significant fluctuations occur in the errors of all directions near the error peaks of the stations, followed by relatively stable variations. After filtering, the error fluctuation patterns become significantly smoother, the range narrows substantially, the amplitude decreases noticeably, and the baseline of the waveform approaches zero. From the changes in RMSE values and 95% confidence intervals before and after filtering for each station, it is also apparent that the filtering algorithm used in this experiment demonstrates remarkable effectiveness in error compensation.

## 4. Discussion

The above experiment demonstrates that, under short-term conditions within a small spatiotemporal range, the positioning errors of low-accuracy GNSS module data exhibit significant variations but follow certain patterns. These patterns can be effectively described using specific curve equations. This modeling approach is simple and efficient, significantly improving the data quality of low-accuracy measurement modules. With this method’s improvement, the accuracy of vast amounts of low-accuracy module data within a region is greatly enhanced, and the data accuracy has improved nearly tenfold. For regional operational spaces, without replacing the equipment, simply processing the received data streams using a filtering algorithm allows these low-cost devices to provide additional information. For instance, in open-pit mine production, low-accuracy modules used for vehicle monitoring can, through slowly moving vehicles, provide massive, real-time updated measurement points for the entire mining area.

In the future, filtered data may serve as a vast source of dynamic control points for updating regional maps.

## Figures and Tables

**Table 1:** Table of changes in RMSE and 95% confidence interval of measurement data before and after filtering at the CUT00 station.

Indicator	Before Filtering	After Filtering	Improvement Percentage
RMSE	5.87	0.92	84.4%
95% Confidence Interval	0.18	0.07	61.5%

**Table 2:** Changes in RMSE and 95% confidence interval of measurement data before and after filtering at the CUTA0 station.

Indicator	Before Filtering	After Filtering	Improvement Percentage
RMSE	11.08	0.46	95.9%
95% Confidence Interval	0.13	0.03	73.2%

**Table 3:** Changes in RMSE and 95% confidence interval of measurement data before and after filtering at the CUTB0 station.

Indicator	Before Filtering	After Filtering	Improvement Percentage
RMSE	6.88	0.82	88%
95% Confidence Interval	0.15	0.06	58.2%

**Table 4:** Changes in RMSE and 95% confidence interval of measurement data before and after filtering at the CUTC0 station.

Indicator	Before Filtering	After Filtering	Improvement Percentage
RMSE	10.39	0.52	95%
95% Confidence Interval	0.11	0.04	67.1%

## Data Availability

The raw data supporting the conclusions of this article will be made available by the authors on request.
